# Knockout of M-LP/Mpv17L, a newly identified atypical PDE, alleviates diabetic conditions in mice

**DOI:** 10.1007/s00592-024-02337-7

**Published:** 2024-07-31

**Authors:** Reiko Iida, Misuzu Ueki, Toshihiro Yasuda

**Affiliations:** 1 Molecular Neuroscience Unit, School of Medical Sciences, University of Fukui, Fukui, 910-1193 Japan; 2 Organization for Life Science Advancement Programs, University of Fukui, Fukui, 910-1193 Japan

**Keywords:** Mpv17-like protein, Cyclic nucleotide phosphodiesterase, Diabetes, Streptozotocin

## Introduction

M-LP/Mpv17L (Mpv17-like protein) was originally found in mouse kidney as a novel gene expressed age-dependently [1]. It has recently been shown that M-LP/Mpv17L is an atypical phosphodiesterase (PDE) that is actually functional in cells despite lacking the conserved catalytic region and other structural motifs characteristic of the PDE family [2]. PDEs are enzymes that degrade cAMP and/or cGMP and are implicated in the regulation of many physiological and pathophysiological processes. cAMP functions as an important second messenger to promote insulin secretion, and pancreatic β-cells express multiple members of the PDE gene family [3]. Among them, PDE1C, PDE3B, and PDE4C are highly expressed and their pharmacological inhibition and/or genetic downregulation is known to enhance glucose-stimulated insulin secretion (GSIS) [4]. Interestingly, mice with knockout (KO) of any of these genes show changes in β-cell mass, whereas mice with KO of the *M-LP/Mpv17L* gene develop pancreatic β-cell hyperplasia as well as improved glucose tolerance, via activation of the Wnt/β-catenin pathway [3]. Since these previous observations have strongly suggested a relationship between M-LP/Mpv17L and diabetes pathology, we conducted experiments to induce type 1 diabetes in wild-type (WT) and *M-LP/Mpv17L*–KO mice by administering streptozotocin (STZ). Here, we report that suppression of M-LP/Mpv17L may alleviate diabetic conditions.

**Fig. 1:**
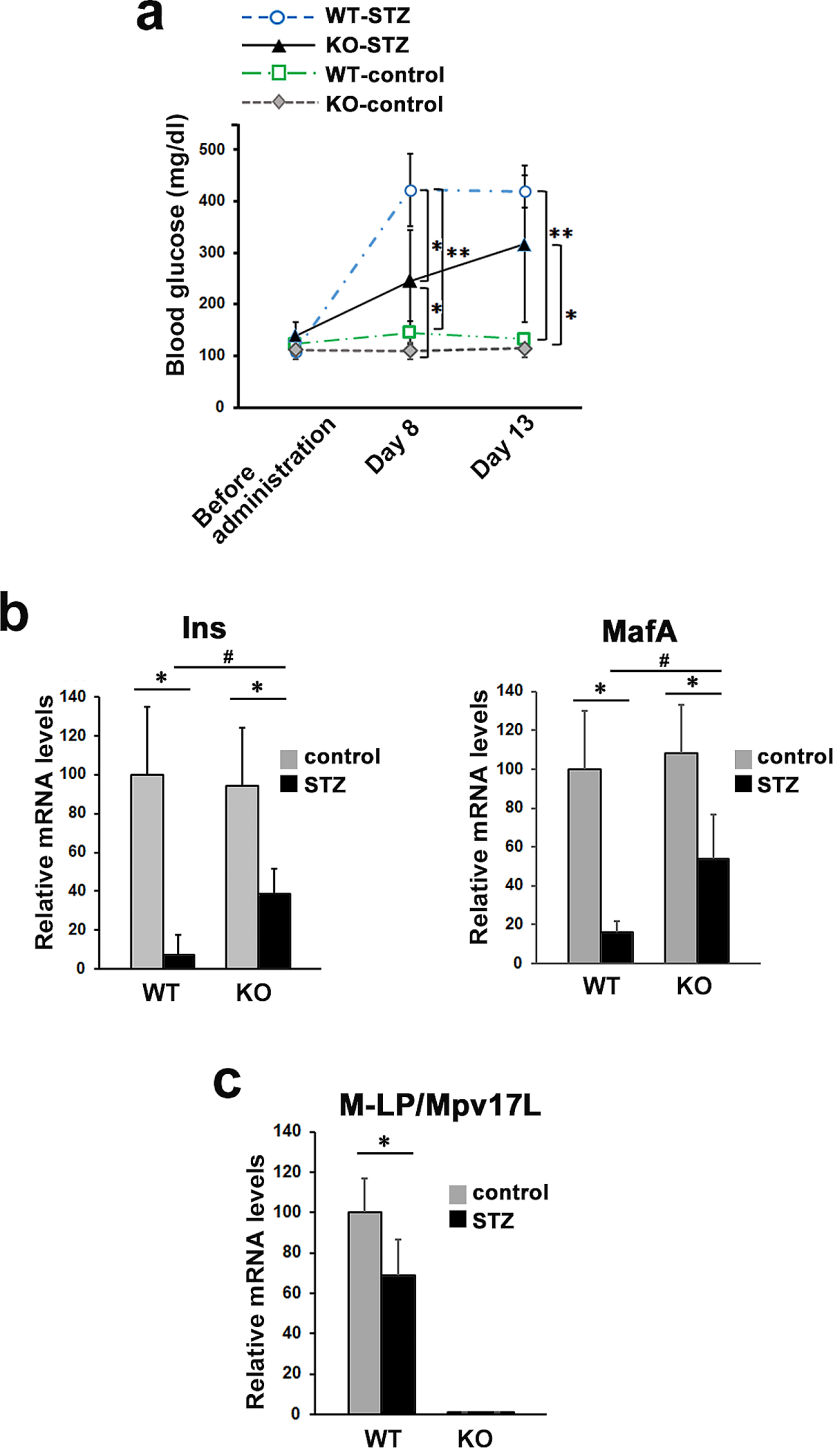
Effects of STZ administration in WT and *M-LP/Mpv17L*-KO mice. a Changes in blood glucose levels in the WT-control, WT-STZ, KO-control and KO-STZ groups (*n* = 5). *p < 0.05, **p < 0.005 b Levels of Ins and MafA mRNAs on day 13 after STZ treatment in the pancreas of the WT-control, WT-STZ, KO-control and KO-STZ groups. Expression of mRNA was determined by Q-PCR using β-actin as an internal control. The results are expressed as ratios relative to the value for the WT-control group (*n* = 4). *p < 0.05, ^#^*p* < 0.05 for similarly treated KO vs. WT. c Levels of M-LP/Mpv17L mRNAs in the pancreas of the WT-control, WT-STZ, KO-control and KO-STZ groups at 13 days after STZ treatment (*n* = 4). M-LP/Mpv17L mRNA was not detected in the KO-control and KO-STZ groups. Expression of mRNA was determined by Q-PCR using β-actin as an internal control. The results are expressed as ratios relative to the value for the WT-control group (*n* = 4). *p < 0.05

**Fig. 2:**
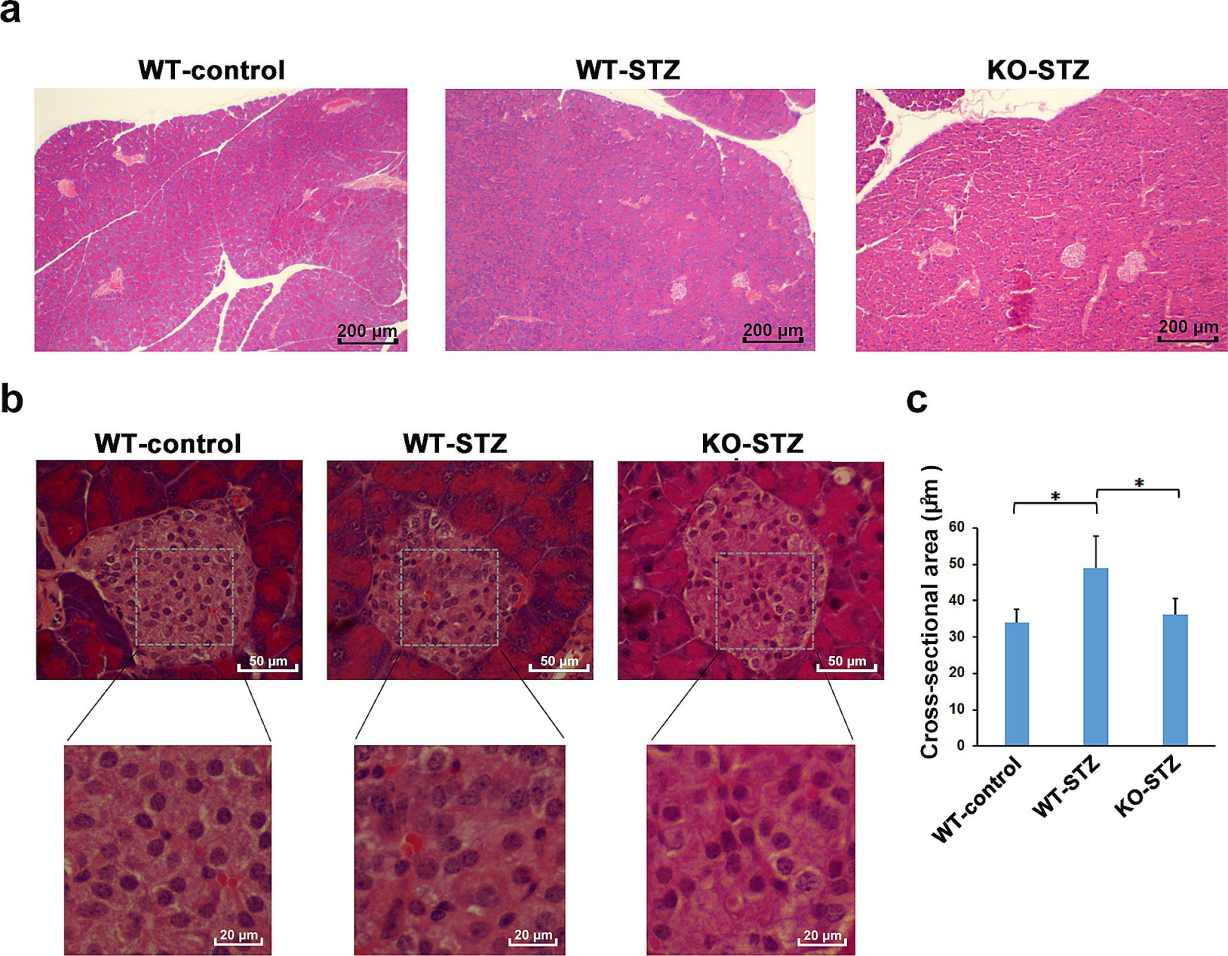
Pathological changes in pancreatic islet cells on day 13 after STZ treatment a, b Representative images of H&E-stained pancreas sections at 13 days after STZ treatment. c Cross-sectional areas of islet cell nuclei in the WT-control, WT-STZ and KO-STZ mice (*n* = 20). *p < 0.005

## Materials and methods

*M-LP/Mpv17L*-KO mice were generated on a C57BL/6 N background as described in our previous paper [3]. The design of the animal experiment was approved by the Committee for Animal Experiments of the University of Fukui. Eight-week-old male WT and *M-LP/Mpv17L*–KO mice were fasted for 18 h before induction of diabetes using STZ (Fujifilm Wako, Osaka, Japan). The WT mice and the *M-LP/Mpv17L-KO* mice were each randomly and equally allocated to treatment with either a single dose of 100 mg/kg STZ dissolved in 0.1 M citrate buffer at pH 4.5 (STZ-treated) or vehicle alone (controls) via intraperitoneal injection. Thus, 4 groups of 5 mice were created: (i) WT-control; (ii) WT + STZ; (iii) KO-control; and (iv) KO + STZ.

Blood glucose levels were measured one day before STZ administration, then on days 8 and 13 after administration using a FreeStyle Freedom Lite (Abbott Laboratories, Abbott Park, IL). Thirteen days after STZ administration, the mice were killed by carbon dioxide inhalation, and the pancreas of each animal was dissected out and fixed in 4% (w/v) paraformaldehyde in phosphate buffer solution for 24 h, then embedded in paraffin for histological analysis. Hematoxylin and eosin (H&E) staining was used for direct microscopic examination. The cross-sectional area of pancreatic islet cell nuclei was measured using the MicroStudio software package (Wraymer, Osaka, Japan).

RNA preparation and quantitative-PCR (Q-PCR) analysis were performed as described in our previous paper [3]. All of the assays were performed at least 3 times, and the results were presented as mean ± S.D. Comparisons between two groups were carried out by unpaired Student’s *t* test. Differences between multiple groups were assessed by one-way ANOVA using GraphPad Prism. *P* values < 0.05 were considered statistically significant.

## Results

Changes in blood glucose levels from the day before STZ administration to days 8 and 13 after administration are shown in Fig. 1a. On day 8, blood glucose level in the WT-STZ group were significantly increased relative to those in the WT-control group (WT-control: 145 ± 21 mg/dl; WT-STZ: 422 ± 70 mg/dl), and high glucose levels in the WT-STZ group were maintained on day 13 after administration (420 ± 31 mg/dl). In contrast, blood glucose levels in the KO-STZ group were higher than those in the KO-control group, but the level on day 8 was significantly lower than that in the WT-STZ group (KO-STZ: day 8, 245 ± 99 mg/dl; day 13, 317 ± 151 mg/dl).

Pancreatic gene expression analysis on day 13 after treatment showed that expression of the β-cell-specific genes, insulin (Ins) and MAF bZIP transcription factor A (MafA), in the KO-STZ group was lower than those in the KO-control group, but significantly higher than those in the WT-STZ group (Fig. 1b). Meanwhile, to examine whether the onset of diabetes caused by STZ administration affected the expression of M-LP/Mpv17L, we analyzed its expression in the pancreas of the WT-control and the WT-STZ groups at 13 days after STZ treatment. This revealed that the expression of M-LP/Mpv17L was significantly down-regulated by STZ treatment (Fig, 1c).

H&E staining of pancreatic Sect. 13 days after treatment showed that, in line with many previous reports, the number and size of pancreatic islets were reduced in the WT-STZ mice relative to the WT-control mice, whereas those in the KO-STZ mice were unchanged (Fig. 2a). Moreover, as shown in Fig. 2b, islets of the WT-STZ mice appeared irregular in shape, with ill-defined borders and nuclear atypia (changes in size and stainability), whereas those of the KO-STZ mice appeared largely intact (Fig. 2b). The cross-sectional area of islet cell nuclei did not differ between the WT-control mice and the KO-STZ mice, but it was significantly increased in the WT-STZ mice (Fig. 2c).

## Discussion

In our previous report, we demonstrated that *M-LP/Mpv17L*-KO mice developed β-cell hyperplasia and improved glucose tolerance, and that the improved glucose tolerance was due to not only the increase in β-cell mass, but also an increase in the insulin secretory ability of β-cells [3]. In addition, this study showed that M-LP/Mpv17L expression was down-regulated in the pancreas of mice with STZ-induced diabetes (Fig. 1c). Interestingly, a recent meta-analysis of datasets consisting of renal samples derived from rat or mouse models of STZ-induced type 1 diabetes and those of type 2 diabetes has recently identified *M-LP/Mpv17L* as one of several shared down-regulated differentially expressed genes (DEGs) in type 1 and type 2 diabetic kidney disease [5]. These observations strongly suggest a relationship between M-LP/Mpv17L and diabetes.

In the present study, deficiency of M-LP/Mpv17L was found to alleviate certain pathological aspects of STZ-induced diabetes such as (1) increased blood glucose levels, (2) down-regulation of β-cell-specific genes, and (3) increased histological changes and damage. These results suggest that suppression of M-LP/Mpv17L expression or activity can alleviate diabetes-associated conditions such as hyperglycemia, and that M-LP/Mpv17L might be a potential target for treatment of diabetes.
